# Supplementary material for: How Does the Gus Schumacher Nutrition Incentive Program Work? A Theory of Change
Source: Nutrients. 2022 May 11;14(10):2018. doi: 10.3390/nu14102018 (PMC9146513; doi:10.3390/nu14102018)
Supplement: Supplementary file 1 [file nutrients-14-02018-s001.zip › nutrients-1665458-supplementary.pdf]

Protocol S1: nutrition incentive evidence review for the Gus Schumacher Nutrition Incentive Program (GusNIP) theory of change

Goal

Identify key references for core theory of change elements as follows:

- Ultimate goals
- Long-term outcomes
- Short-term outcomes
- Activities
- Environmental context
- Assumptions

Questions explored

- What are the impacts of nutrition incentive projects on participants, participating food retailers, and communities?
- What do people participating in nutrition incentive projects need to be able to successfully use nutrition incentives?
- What are nutrition incentive program design features that are associated with increased fruit and vegetable purchases, consumption, and food security for participants?
- What are the nutrition incentive program design features that facilitate food retailers participation in nutrition incentive projects?

Inclusion criteria

- *Publication type:* We included peer-reviewed articles and gray literature reports. We excluded abstracts, books, and conference proceedings.
- *Population:* Human participants of any age.
- *Language:* English only.
- *Study Design:* We included the following experimental study designs: controlled trial or other comparative study design (e.g., randomized controlled trial, controlled trial or quasi-experiment, or natural experiment); single-group pre/post or post-only; and time series. We also included qualitative, systematic and other reviews; meta-analyses; cross-sectional designs; and simulation or modelling studies.
- *Setting:* Any country. Farm direct and brick and mortar food retail settings.
- *Nature of Intervention*: Any type of healthy food pricing incentive strategy. We also included studies that combined multiple interventions, if increasing access to healthy foods via a healthy food pricing incentive was a primary component of the intervention; excluded produce prescription projects.
- *Date Range:* 1/1/18 – 7/2/21 (period prior to 1/1/18 covered by Healthy Food America review[1]).

Databases, search date, search terms and selection

Table S1. Databases, search dates, search terms, and selection

Source	Date(s) of search	Search Terms	Selection
PubMed	November 20, 2020<br>January 8, 2021<br>July 2, 2021	See search hedge below.	KL conducted the search and reviewed article titles and abstracts to determine article inclusion. KL conducted a full text screen of the selected articles and identified articles for full text extraction. KL extracted data and JK reviewed to determine final inclusion status.
Google Scholar	June 2021<br>December 2021	SNAP participants barriers fruit and vegetable consumption<br>(FINI OR GusNIP OR nutrition incentives) AND implementation AND (stores OR participants)<br>SNAP store environment<br>SNAP incentive retailer environment<br>Store implementation GusNIP<br>(supermarket OR farmer market OR grocery store) AND (SNAP OR nutrition incentive)<br>(farmer) AND (SNAP OR nutrition incentives)	KL conducted the search and reviewed titles and abstracts for articles retrieved on the first two pages of results to determine eligible articles. KL conducted a full text screen of the selected articles and identified articles for full text extraction. KL extracted data and JK reviewed to determine final inclusion status.

Gray literature sources: Gretchen Swanson Center for Nutrition website Fair Food Network website The Food Trust website Farmers Market Coalition website	April 2021	Not applicable	KL reviewed key nutrition incentive organization websites to identify relevant gray literature reports.
Identifying Additional Articles: Nutrition incentive experts	August 2020 – November 2021	Not applicable	Additional articles were contributed by the authors and TOC contributors. KL conducted a full text screen of the selected articles and identified articles for full text extraction. KL extracted data and JK reviewed to determine final inclusion status.

Data Extraction

A data extraction tool was developed for use in Excel. Data extraction variables included:

- Article citation
- Abstract
- Key findings
- Setting (farmers market, brick & mortar, food retailer, online, mobile market)
- Corresponding component of theory of change (e.g., ultimate goal, long-term outcomes, etc.)

PubMed Search Hedge

(“Consumer Behavior” [mesh] OR “Consumer Behavior” [tiab] OR “Choice Behavior”[tiab] OR “Commerce”[mesh] OR Purchase*[tiab] OR “purchasing”[tiab] OR buying[tiab] OR buy[tiab] OR bought[tiab] OR pay[tiab] OR payment*[tiab] OR sales[tiab] OR sale[tiab] OR selling[tiab] OR Consumer*[tiab] OR Retail*[tiab] OR Market[tiab] OR markets[tiab] OR store[tiab] OR stores[tiab] OR supermarket*[tiab] OR grocery[tiab] OR groceries[tiab] OR “food outlet”[tiab] OR “food outlets”[tiab] OR school[tiab] OR schools[tiab] OR worksite*[tiab] OR workplace*[tiab] OR “work site”[tiab] OR “work sites”[tiab] OR “work place”[tiab] OR “work places”[tiab] OR cafeteria*[tiab] OR “concession stand”[tiab] OR “concession stands”[tiab] OR “restaurant”[tiab] OR “restaurants”[tiab])
AND
(“Diet” [mesh] OR “Diet” [tiab] OR “diets” [tiab] OR “dietary” [tiab] OR “Healthy Diet” [mesh] OR (food[tiab] NOT ("food and drug"[tiab] OR "food safety"[tiab] OR "food production"[tiab])) OR foods[tiab] OR “Food”[mesh] OR “Eating” [mesh] OR “Eating” [tiab] OR nutritious[tiab] OR “Food Preferences” [mesh] OR "Diet, Food, and Nutrition"[Mesh:NoExp] OR “Food and Beverages” [mesh] OR beverage*[tiab] OR “Beverages” [mesh] OR fruit[tiab] OR fruits[tiab] OR vegetable[tiab] OR vegetables[tiab] OR "Food Quality"[Mesh:NoExp] OR "Nutritive Value"[Mesh])
AND
(“Reward” [mesh] OR “Reward” [tiab] OR “Rewards” [tiab] OR “incentive” [tiab] OR “incentives” [tiab] OR “reimbursements” [tiab] OR “reimbursement” [tiab] OR “financial” [tiab] OR “monetary” [tiab] OR “price” [tiab] OR “prices” [tiab] OR “pricing” [tiab] OR priced[tiab] OR costs[ti] OR cost[ti] OR “promotions” [tiab] OR “promotion”[tiab] OR “discount” [tiab] OR “discounts” [tiab] OR “discounted”[tiab] OR “voucher” [tiab] OR “vouchers” [tiab] OR “coupon” [tiab] OR “coupons” [tiab] OR “subsidy” [tiab] OR “subsidies” [tiab] OR subsidized[tiab] OR “rebate” [tiab] OR “rebates” [tiab] OR “cash” [tiab] OR “money” [tiab] OR “food dollars”[tiab] OR “supplemental nutrition assistance program”[tiab] OR wic[tiab] OR "Food Assistance"[Mesh] OR "Public Assistance"[Mesh:NoExp])
AND
(“Clinical Studies as Topic” [Mesh] OR “clinical study” [Publication Type] OR “Controlled Before-After Studies” [mesh] OR “historically controlled study” [mesh] OR “interrupted time series analysis” [mesh] OR “comparative study” [pt] OR “research design” [mesh] OR ((clinical[tiab] OR controlled[tiab]) AND (study[tiab] OR studies[tiab] OR trial*[tiab])) OR randomi*[tiab] OR “time series”[tiab] OR “systematic review”[tiab] OR “systematic reviews”[tiab] OR “meta-analysis”[pt] OR metaanalysis*[tiab] OR “meta analysis”[tiab] OR “Meta-Analysis as Topic”[Mesh] OR “Review Literature as Topic”[Mesh] OR “Review” [Publication Type] OR intervention*[ti] OR “model” [tiab] or “modeling” [tiab] OR “modelling” [tiab] OR models, theoretical [mesh] OR models, theoretical [tiab] OR “economic intervention”[tiab] OR “economic interventions”[tiab] OR “effectiveness” [ti] OR "Validation Studies" [Publication Type] OR "Comparative Effectiveness Research"[Mesh] OR “program evaluation” [mesh] OR “evaluation studies as topic” [mesh] OR “evaluation studies” [publication type])
AND
English[la]
AND
(“2000”[Date - Publication]: “3000”[Date - Publication])
NOT
(animals[mesh] NOT humans[mesh]

Moderator Guide S1: Partner and practitioner interviews

Introduction Script:

Thank you for taking the time to speak with me today. My name is __ and I am a Research Consultant for Healthy Food America, a nonprofit nutrition policy and research organization. We are working with the Gretchen Swanson Center for Nutrition to develop a theory of change (TOC) for the Gus Schumacher Nutrition Incentive Program (GusNIP). We are interviewing you today as part of an information-gathering process that will help us draft the TOC.

Before we dive into the questions, I want to briefly touch on what a TOC is – my hope is this will provide some context for the questions I have for you today. [Share TOC diagram with definitions]. A TOC describes how and why an initiative is expected to produce a desired change. It identifies the goal or goals of an initiative and maps out what needs to happen for the goal to be achieved and the specific activities that are needed along the way. This diagram represents a very bare bones TOC [explain diagram]. It is not necessary for you to have a deep understanding of TOC for our conversation today, but before we move on, do you have any questions about TOC generally?

The interviews are confidential. There are no right or wrong answers. Your perceptions and experiences with GusNIP are needed to help us develop a TOC that maps out how GusNIP works so please share based on your understandings.

Just a few final details before we get started. I will be taking notes during our conversation today and also recording our conversation (for note taking purposes only). Is that OK? No formal report specific to these interviews will be developed but the compiled learnings from the interviews may be used to inform a peer-reviewed publication/ paper in the future. The draft TOC will be shared with you and other stakeholders in a few months with more opportunity to comment.

Our conversation should last about an (hour/ hour and a half). Do you have any questions or concerns before we begin? Great. Let's get started.

Question Block 1. Interviewees

Let's start with some brief introductions.

1. Could you please tell me a little about yourself and your work related to food incentives/ the GusNIP initiative?

For the next several questions I will be capturing your thoughts on the screen so please correct me if I get anything wrong.

Question Block 2. GusNIP Goals

These first questions focus on what GusNIP is ultimately trying to achieve. In other words, what are the goals or the impacts that will be reached over the long-term?

1. Tell me about the goals of the GusNIP initiative.

    a. Probe: What are the overarching things GusNIP is trying to accomplish? Some examples might be increasing FV consumption, increasing food security, supporting local food systems, or supporting the local economy.
2. (If name more than 3): Of these goals, which two or three are most important?
    a. Probe: Tell me more about what makes these important?
3. (If none of the GSCN goals surface). There are a few other goals we've heard about. We'd like to know what you think about them:
    - Improved FV consumption
    - Improved food security
    - Economic benefits (local economy, farmers, etc.)
    - Improved health outcomes

Question Block 3. Outcomes

Now we're going to look at each of these goals one at a time and explore the things that have to happen on the way to reaching the goal. These are things like changes to systems, knowledge, and processes. There are likely several steps that have to happen on the way to reaching the goal. For example, before we can reach the goal of increasing FV consumption, participants need to use the incentives to purchase the FV, before that can happen, participants need to visit a retail site that offers incentives, before that can happen participants must know about and have access to these retail sites, etc.

4. What needs to exist/ be accomplished for goal [1] to be reached? (If needed, clarify there can be multiple paths that lead to the goal).
    a. Probe: How do you think these steps lead to the goal/ "outcome" above it?
    b. Probe: [For each outcome clarify what needs to happen before this outcome can be achieved?]

Question Block 4. Activities

Great now that we've identified what needs to happen to reach this goal, let's shift to discuss the specific activities you've been involved with under GusNIP/ with nutrition incentives that help to bring about these "outcomes". For example, if the outcome is participants learn about incentives, activities might be SNAP incentive signage at the point of purchase or education during a community class. If the outcome is farmers markets can process nutrition incentives, an activity might be around technical assistance

5. Tell me about the activities you've been doing or are planning to do as part of GusNIP/ with nutrition incentives?
6. How do you think these specific activities produce the outcomes we just discussed?
    a. Probe: [For each activity, ask about which outcome(s) it's related to and why they think it will produce that outcome].
    b. (If time allows) Probe: [Is this based on data? Research? Experience? Logic? Assumptions?]

[Repeat Question Block 3 and 4 for Goal 2. Repeat for Goal 3 as time allows]

Question Block 5. Equity

These next questions aim to explore how GusNIP contributes to equity. For our purposes today, when we say equity we mean assuring access to healthy food for people at risk of food insecurity based on race,

ethnicity, geography, SES, etc. and that those impacted by food insecurity are involved in identifying root causes, suggesting solutions and participating in implementing responses/ solutions.

7. Tell me about how what you are doing through GusNIP impacts equity?
    a. Probe: For example, how are you identifying people affected by lack of access to healthy food and centering this program on them?
8. (If time allows) What specific outcomes or activities are likely to promote equity?
9. (If time allows) Do you have any concerns that any of the GusNIP outcomes or activities might decrease equity, even if it is unintended?
10. (If time allows) What challenges still exist that prevent your work from producing equitable outcomes/goals?

Question Block 8. Closing

Great – thanks so much for sharing all of this with me today. In closing out our conversation today,

11. Do you have any additional thoughts that you would like to share?

Closing Script

Thank you again for your time today. We will be sharing a draft of the TOC with opportunity to comment in the beginning of the new year. Please feel free to reach out with any questions or additional feedback in the meantime.

Moderator Guide S2: Participant interviews

Introduction Script:

Thank you for taking the time to speak with me today. My name is ___ and I work for an organization called Healthy Food America in Seattle. We're working on a project to learn more about how fruit and vegetable (FV) vouchers work. We're interested in knowing what makes them easy to use and what is hard about using them. We are talking to you today to help us figure this out.

A few things before we get started. Our conversation today is confidential. There are no right or wrong answers. Please just share based on your ideas about and experiences with fruit and vegetable vouchers. I would like to take notes during our conversation today. Is that OK?

Our conversation should last about an hour. Do you have any questions or concerns before we begin? Great. Let's get started.

Question Block 1. GusNIP Goals.

1. What made you interested in getting FV vouchers?
2. Tell me what you like about FV vouchers?
    a. Probe: How have incentives helped you and your family?
        i. Are they helpful for…:
            1. Getting healthier food? (How?)
            2. Making food more affordable? (How?)
            3. Having enough money to buy food for your family? (How?)
            4. Improving your health and the health of family members (How?)
            5. Making it possible to buy food from famers at farmers markets? (How?)
3. Tell me what you don't like about FV vouchers?

Question Block 2. Outcomes and Activities

Now I'd like to learn about what it takes to be a part of the FV voucher program and use the vouchers.

4. Think about your experience of using FV Vouchers. Tell me about the steps you took to be able to use the vouchers? [Take notes on their process, then review each step and ask what worked well and what made that step hard. Use questions below to guide and fill in gaps as needed].
    a. How did you first learn about FV vouchers?
        i. Are there other ways to let people know about them?
    b. How did you first sign-up for the program?
        i. What made it easy to sign-up?
        ii. What made it hard to sign-up?
            1. Are there things that would have made it easier to sign-up?
    c. How do you get your vouchers?
        i. What made this a good way to get them?
        ii. Are there other ways that would have made it easier?
    d. Where do you go to use your vouchers?
        i. Why did you choose this /these place(s)?
            1. What makes it easy to use your vouchers here?
            2. What makes it hard for you to use your vouchers here?
        ii. Are there other places you'd like to able to use your vouchers?

e. How do you use the food you get with the vouchers?
    i. Are there challenges for you or your family in eating or preparing the FV?
        1. Would information/tips on how to cook what you get be useful?
        2. Is it hard to find the time to prepare them?
        3. Does your family like them?
        4. Do you have what you need in your kitchen to prepare them?

Question Block 4. Closing

Great – thanks so much for sharing all of this with me today. In closing,

5. Do you have any additional thoughts that you would like to share?

Closing Script

Thank you again for your time today. As a small token of our appreciation and to honor the time you have spent with me today we would like to offer you a $50 visa gift card. Do you prefer to receive this electronically or have one mailed to you? [Record email or mailing address].

Agenda S1: Workshops

Session One

February 1, 2020
11am-1pm CT / 9am-11am PT

Workshop Objectives:

- Understand what a TOC is (Session 1)
- Contribute to development of a TOC for GusNIP (Session 1, Session 2, Session 3)
- Ensure an equity lens is applied to the GusNIP TOC (Session 3)

11:00 – 11:15	Welcome, Introductions & Ice Breaker<br>Name, relationship to GusNIP, what excites you about GusNIP
11:15 – 11:30	TOC Project & Workshop Overview<br>Agenda & Ground Rules<br>What is a TOC?<br>GusNIP TOC Process
11:30 – 11:40	Why develop a TOC for GusNIP?<br>Brainstorm: How will you use the GusNIP TOC?
11:40 – 11:55	GusNIP TOC Overview<br>Orientation to current GusNIP draft TOC
11:55 – 12:35	Breakout Session: GusNIP TOC Goals<br>Are the identified goals accurate?<br>Are there goals that are missing?<br>Should any goals be modified?<br>Should any goals be deleted?
12:35 – 12:55	GusNIP TOC Outcomes<br>Overview of TOC outcomes pathways<br>Discussion: Initial reactions or questions
12:55 – 1:00	Closing & Homework<br>Review outcomes pathways<br>What’s missing? What should be modified? Removed?<br>What are the activities you do to make these outcomes happen?

Session Two

February 5, 2021
1:30 – 4:00 pm CT / 11:30 am-2:00 pm PT

Workshop Objectives:

- Understand what a TOC is (Session 1)
- Contribute to development of a TOC for GusNIP (Session 1, Session 2, Session 3)
- Ensure an equity lens is applied to the GusNIP TOC (Session 3)

1:30 – 1:40	Welcome & Introductions<br>Name, one word to describe your week
1:40 – 2:00	Summary of Session One<br>Review main points of discussion from session one<br>Any changes? Anything missing?
2:00 -3:00	Breakout Session Core TOC Outcomes<br>Are the identified outcomes well described? Is any re-wording needed?<br>What outcomes are missing?<br>Are there any outcomes that don't belong?<br>Are the outcomes pathways in the right places and in the right order?<br>What is the evidence/ rationale for how outcomes lead to other outcomes or goals?
3:00 – 3:50	Breakout Session Foundation & Evaluation/ Sustainability Outcomes<br>Are the identified outcomes well described? Is any re-wording needed?<br>What outcomes are missing?<br>Are there any outcomes that don't belong?<br>Are the outcomes pathways in the right places and in the right order?
3:50 – 4:00	Closing & Homework<br>Homework:<br>Equity – How might the TOC goals and outcomes be modified to better promote equity? Are there goals and outcomes that are unintentionally negatively impacting equity?<br>Environmental Context / Assumptions – What are assumptions about GusNIP? What external factors affect GusNIP? Which of these might be included in a GusNIP TOC 2.0?<br>Next Session: Friday, 2/12/21 10:00 am – 12:30 pm CT

Session Three

February 12, 2021
10:00 am – 12:30 pm CT / 8:00 am - 10:30 am PT

Workshop Objectives:

- Understand what a TOC is (Session 1)
- Contribute to development of a TOC for GusNIP (Session 1, Session 2, Session 3)
- Ensure an equity lens is applied to the GusNIP TOC (Session 3)

10:00 – 10:10	Welcome & Introductions<br>Name, organization, favorite COVID silver lining
10:10 – 10:30	Agenda Review & Summary of Session Two<br>Review main points of discussion from session two<br>Any changes? Anything missing?
10:30 – 11:30	Breakout Session Applying an equity lens to the GusNIP TOC 1.0<br>Review equity TOC discussion points from interviews and workshop sessions one and two<br>Do the goals and outcomes accurately describe how the current GusNIP program impacts equity? Which promote equity? Which may have the unintended consequence of increasing inequity?
11:30 – 12:20	Breakout Session Environmental Context & Underlying Assumptions<br>Review context and assumptions shared during interviews and workshop sessions one and two<br>What's missing from this list? Does anything need to be modified? Elaborated? Clarified?
12:20 – 12:30	Closing<br>Additional feedback and workshop evaluation<br>Next steps: TOC diagram revisions and narrative development<br>Workshop reflections<br>Thank you!

Moderator Guide S3: Focus groups

Welcome and Overview

- Welcome everyone! Thanks for taking the time to join me today to talk about NI [Use local program name for SNAP nutrition incentives for FV]. My name is ____ and I work for _____. We're interested in learning how you use NI and how they help you and your family. We are having conversations like this one with other groups across the country. We can then use what we learn from these conversations to better understand how these types of programs work and what is needed to make them work well.
- We will spend the next [75-90 minutes] talking about NI. I'll be asking you all questions to discuss as a group. We have a lot to talk about today, so I'll also help keep our conversation on track.
- You were each invited because you participate in the ____ program so you're familiar with NI. Keep in mind that there are no right or wrong ideas here. We want to hear about your experiences and opinions. Please feel free to share your point of view even if it is different from what others have said. We're just as interested in negative comments as positive comments, and at times the negative comments are the most helpful. Finally, I want to make sure that we hear from everyone today, so I'll be encouraging each of you to participate.
- To make sure everyone is able to share their opinions, it would be great if we could all agree that only one person will speak at a time. If you feel like you are having a hard time sharing your thoughts, you can click on the "chat" icon at the bottom of your screen to send a message to everyone. You can also click on the "reactions" icon at the bottom of your screen to "raise your hand" if you have something to say. You don't have to use these features though, only if they are helpful.
- I would also like to record this conversation for note taking purposes only. I don't want to miss any of your comments. People often say very helpful things in these discussions, and I can't write fast enough to get them all down. All responses are confidential, and no names will be used in reporting. Is it OK for me record? [Start recording]
- We'll start with some introductions, but before we do, does anyone have any questions?

Introductions

Great, let's take a minute for introductions. If you could each share your first name, your favorite fruit or vegetable and how you like to eat it or cook it? [Moderator facilitate round robin of participant self-introduction]

NI Process Questions (Core TOC Outcomes)

It's so nice to meet everyone. Let's get started with our questions.

1. Let's say someone new moved to your community. This new neighbor is having a hard time buying FV for her family. What would you tell her about the NI program and how the NI program works?

   [Moderator asks for a volunteer to start. Encourage everyone to share.]

   Probes:

- Would anyone share anything differently?
- Is that the same for you? Or is there something different?
- [If anything needs more clarification] Tell me more about ___.

2. Now let's discuss your experiences with NI. What are the steps in the NI process? Please walk us through the process from start to finish. You can tell us about all the key steps: how you first heard about the NI program, how you got the NI, where you used the NI, what items you purchased with the NI, and how your household used the items you purchased with NI.

   [Moderator asks for a volunteer to start]:

   Probes:

   - [For any of the steps that need more clarification] Tell me more about ___.
   - Has anyone had a different experience?
   - Is that the same for you? Or is there something different?
   - Other people have said ___. What do you think?
   - Walk me through what it's like to ___.

[Steps trying to address. If any of these steps don't come up, moderator should probe]:

a. Please tell me about how you first learned about NI? Tell me more about what you learned? [If time allows: What other information would have been helpful to know when you first learned about NI? Are there other ways to let people know about NI?] (7)
b. Tell me about how you signed up for NI? [If time allows: Are there other ways that would have made it easier?] (9)
c. Tell me about how you get your NI vouchers/tokens/etc.? [If time allows: What made this a good way to get them? Are there other ways that would have made it better?] (9)
d. Where do you go to use your NI? How did you find out about these stores/ markets? Tell me about how you get there [probe: Easy? Hard?]? How did you decide to shop there? What makes it easy/ hard to use your NI there (examples: transportation, store hours convenient, technology used to process NI easy, good customer experience, etc.)? Tell me about how these stores/ markets reflect (or don't reflect) your community? Tell me about other places you'd like to be able to use your NI? (13, 16, 18, 19, 20, 22).
e. How important is it to you to eat local FV? Where do you go to use your NI to get local FV? [If site not covered in "d": Tell me about using your NI there]. (4, 10, 28, 29)
f. There are a lot of reasons why people choose certain fruits and vegetables. Can you tell me about the fruits and vegetables you choose? [Examples if needed: cost, quality, FV from your culture, FV that are easy to prepare]. (13)
g. Walk me through how you use the FV when you get home. [Probes: who eats, who cooks, challenges in preparation -time, resources, skills, preferences]. (1, 2, 3)
h. Do you and your family like eating fruits? Vegetables? Do you eat enough FV, or would you like to eat more? If you want more, what gets in the way of doing so [examples: cost, can't find them in stores I go to, etc.]? (8, 10,11)]

NI Benefits Questions (TOC Goals)

3. [Moderator – the answer to this question may have already been discussed by this point] What made you interested in getting NI/ What do you like about NI? [probes: does it make it easier for you to eat more FV? Improve health? Help financially?]

[If time allows] Community Participation Questions (Foundation and Evaluation)

4. When community programs like NI are being developed, it's really helpful to have feedback from program participants (like you all) to be sure the program is going to work. Tell me about some ways you and other people in the community could help set up these programs [probes: participate in focus groups, active members on advisory councils, consider employment with program, etc.].

[If time allows] GusNIP TOC 2.0

5. We've talked a lot about how the program currently works and some things that could be done to make it work better. What other things would you change to make it work better?

Ending Questions

Thank you. I really value all that you each have shared today. We only have __ minutes left, as we wrap up the conversation today:

6. Of all the things we've talked about, what is most important to you? [Moderator facilitate round robin feedback]
7. Does anyone have any final comments to share before we wrap up?

Thank you again for spending time with me today. I really enjoyed our conversation. We will be sharing this feedback with people who run nutrition incentive programs to better understand how these programs are used. As ___ [site coordinator] shared, you all will be receiving a $75 gift card for your time today. You will receive it by __[explain stipend process].

References

1. Healthy Food America. Healthy Food Pricing Incentives: A systematic review of current evidence. 2019. https://www.healthyfoodamerica.org/healthy_food_pricing_incentives_a_systematic_review_of_current_evidence
